# Polymorphous low-grade neuroepithelial tumor of the young: a case report

**DOI:** 10.3389/fonc.2026.1802067

**Published:** 2026-06-05

**Authors:** Haitao Wu, Youyuan Chen, Peng Sun, Wenshuai Deng

**Affiliations:** 1 Qingdao University Medical College, Qingdao, China; 2 Department of Neurosurgery, The Affiliated Hospital of Qingdao University, Qingdao, Shandong, China

**Keywords:** BRAF V600E, imaging, long-term epilepsy-associated, pathology, polymorphous low-grade neuroepithelial tumor of the young (PLNTY)

## Abstract

**Objective:**

To investigate the clinical, imaging, pathological, and molecular characteristics of polymorphous low-grade neuroepithelial tumor of the young (PLNTY) and provide references for clinical diagnosis and treatment.

**Method:**

We retrospectively analyzed the clinical data of one pathologically confirmed PLNTY patient, including medical history, clinical symptoms, imaging findings, pathological morphological features, immunohistochemical results and molecular testing data, and reviewed relevant literature.

**Result:**

A 30-year-old male patient presented with a 6-year history of drug-resistant epilepsy as the primary manifestation, which progressed from focal facial myoclonus to mixed seizure phenotypes. Cranial magnetic resonance imaging (MRI) demonstrated an ovoid area of abnormal signal intensity in the left temporal pole, exhibiting isointensity on T1-weighted imaging (T1WI), hypointensity on T2-weighted imaging (T2WI) and fluid-attenuated inversion recovery (FLAIR) sequences, and no enhancement following contrast administration; the initial radiological impression was a suspected cavernous hemangioma. Twenty-four-hour video electroencephalography (vEEG) identified abnormal epileptiform discharges originating from the left anterior temporal lobe. Subsequent to left temporal lobe lesionectomy, pathological examination confirmed the diagnosis of polymorphous low-grade neuroepithelial tumor of the young (PLNTY), CNS WHO Grade 1. Microscopically, the lesion was characterized by thin-walled vascular clusters, focal vascular wall hyalinization, calcification, and adjacent reactive glial proliferation. Immunohistochemical (IHC) staining revealed diffuse CD34 positivity, glial fibrillary acidic protein (GFAP) (+), oligodendrocyte transcription factor 2 (Olig-2) (+), and a Ki-67 proliferation index of 1%, indicating low proliferative activity. Molecular testing confirmed BRAF mutation positivity, weak immunoreactivity for BRAF V600E (detected via VE1 antibody), and absence of malignant-associated mutations. Postoperatively, the patient achieved complete seizure control and remained seizure-free with uneventful recovery at the latest follow-up.

**Conclusion:**

PLNTY is typically characterized by long-standing drug-resistant epilepsy and has a predilection for the temporal lobe, with potential atypical imaging manifestations (e.g., hypointensity on T2WI as observed in this case). Definitive diagnosis requires the integration of pathological morphology, immunohistochemistry, and molecular testing. PLNTY harboring the BRAF V600E mutation exhibits indolent biological behavior; gross total resection serves as the core therapeutic modality, conferring a favorable prognosis. Molecular subtyping can guide individualized follow-up strategies, and clinicians should be vigilant against misdiagnosis of cases with atypical imaging features.

## Background

Polymorphous low-grade neuroepithelial tumor of the young (PLNTY) is a distinct type of epileptogenic neuroepithelial tumor, first described by Jason T. Huse et al. in 2016 (1), and subsequently classified as a subtype of pediatric-type diffuse low-grade glioma in the 2021 World Health Organization (WHO) Classification of Tumors of the Central Nervous System (2). This tumor predominantly affects adolescents, with drug-resistant epilepsy as its core clinical feature. Most patients develop symptoms during adolescence and often have a long-standing history of drug-resistant epilepsy prior to definitive diagnosis. PLNTY lacks specific radiological manifestations and characteristic diagnostic signs. Following gross total resection of the lesion, the frequency of epileptic seizures is significantly reduced or even completely controlled in most patients, with favorable postoperative prognosis. To date, fewer than 100 cases of PLNTY have been reported worldwide, and the current understanding of this tumor remains incomplete. Herein, we report a pathologically confirmed case of PLNTY, systematically analyze its clinical, radiological, and pathological features, and review relevant literature, aiming to provide references for clinical diagnosis and treatment.

## Patient information

A 30-year-old male patient presented with occasional nocturnal facial muscle twitching without obvious predisposing factors 6 years prior to admission; the episodes occurred without loss of consciousness and spontaneously resolved. He was diagnosed with epilepsy at an outside hospital and received standard oral oxcarbazepine treatment. Since 2022, his epileptic symptoms had exacerbated, with the frequency of generalized tonic-clonic seizures increasing to once monthly, accompanied by daily minor episodes characterized by several seconds of absence. Continued treatment with the original regimen resulted in poor symptom control, prompting him to visit our hospital for further diagnosis and treatment.

Upon admission, cranial magnetic resonance imaging (MRI) was performed, revealing an ovoid abnormal signal lesion in the left temporal pole. The lesion showed isointensity on T1-weighted imaging (T1WI), hypointensity on T2-weighted imaging (**Figure 1A**) and T2 fluid-attenuated inversion recovery(**Figure 1B**) sequences, and isointensity on diffusion-weighted imaging (DWI), with a diameter of approximately 8 mm. No abnormal enhancement was observed on contrast-enhanced scan. Initial imaging diagnosis suggested a possible cavernous hemangioma in the left temporal pole region.

**Figure 1:**
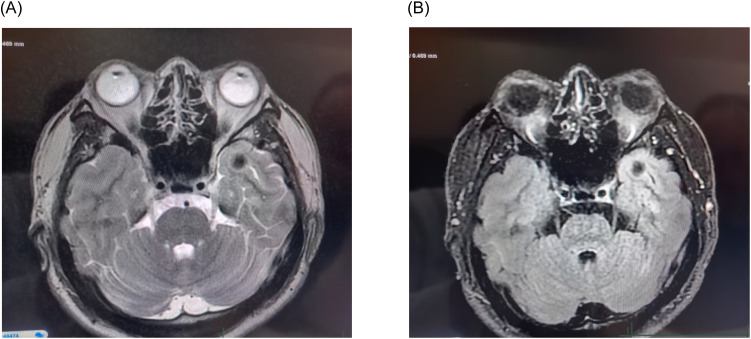
Cranial MRI findings. **(A)** Axial T2-weighted image showing a small ovoid hypointense lesion in the left temporal pole. **(B)** Axial T2 fluid-attenuated inversion recovery (FLAIR) image showing corresponding hypointensity of the lesion. The lesion measured approximately 8 mm in diameter, and no abnormal enhancement was observed on contrast-enhanced MRI (not shown).

Concurrently, 24-hour video polysomnography-electroencephalography (EEG) monitoring was conducted(**Figure 2**), and the results indicated that abnormal discharges originated from the left anterior temporal lobe, involving the frontal pole and sphenoidal electrode regions. The evolution of electroencephalographic patterns during seizure episodes was consistent with the characteristics of focal epileptic seizures.

**Figure 2:**
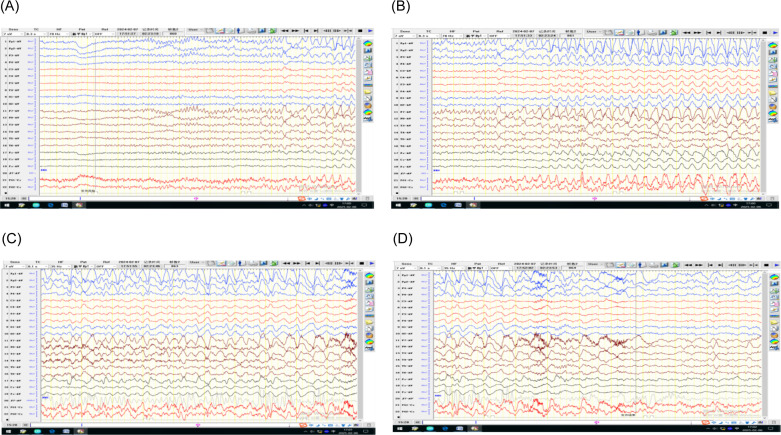
Twenty-four-hour video electroencephalography (vEEG) findings. **(A–D)** Sequential EEG screenshots showing abnormal epileptiform discharges originating from the left anterior temporal lobe and involving the frontal pole and sphenoidal electrode regions. The electroencephalographic evolution during the seizure episode was consistent with focal epileptic seizures.

Based on the patient’s medical history, imaging findings, and electroencephalography results, left temporal lobe lesionectomy was performed on November 27, 2024. From a surgical perspective, the concordance between the small calcified lesion in the left temporal pole and epileptiform discharges from the left anterior temporal region supported a lesion-directed resection strategy. Intraoperatively, the lesion appeared as a grayish-white, firm mass. Owing to extensive calcification, the boundary between the lesion and the surrounding brain parenchyma was relatively well defined. No obvious perilesional edema, abnormal neovascularization, or superficial hemosiderin deposition was observed. Although the lesion was radiologically suspected to be a cavernous malformation, these intraoperative findings were not typical of a classic cavernous malformation. Surgical removal was therefore considered necessary not only for seizure control but also for obtaining a definitive histopathological and molecular diagnosis.

Postoperative pathological examination revealed brain cortex and white matter structures with focal glial proliferation, extensive calcification, mild cytological atypia, oval-to-spindle-shaped tumor cells, and focal small-vessel proliferation. Immunohistochemical staining showed GFAP positivity, Olig-2 positivity, IDH-1 R132H negativity, wild-type p53 expression, retained ATRX, negative H3K27M, retained H3K27me3 and MTAP expression, CD34 positivity with diffuse expression in the lesion, weak BRAF V600E VE1 immunoreactivity, and a low Ki-67 proliferation index of <1%. Based on the integrated histopathological and immunohistochemical findings, the final pathological diagnosis was polymorphous low-grade neuroepithelial tumor of the young, CNS WHO Grade 1. DNA methylation profiling was not performed in this case.

The patient received regular postoperative follow-up, and no epileptic seizures were recorded by the last follow-up, with satisfactory recovery.

## Discussion

As a newly classified subtype of pediatric-type diffuse low-grade glioma in the 2021 WHO Classification of Tumors of the Central Nervous System, PLNTY is characterized by a strong association with long-term epilepsy and is prone to misdiagnosis in clinical practice due to non-specific radiological manifestations. The 30-year-old male patient reported in this study exhibits both the typical clinicopathological features and unique radiological manifestations of PLNTY throughout his disease course and diagnostic-therapeutic process, which will be discussed below in conjunction with relevant literature.

PLNTY is now widely acknowledged as a distinct entity among long-term epilepsy-associated tumors (LEATs) (3), with a striking predilection for supratentorial locations. It most commonly involves the temporal lobe, followed in frequency by the frontal and parietal lobes, and only rarely affects the ventricular system (4). Predominantly afflicting pediatric and adolescent populations, PLNTY classically manifests with seizures secondary to an epileptogenic mass lesion or, far less commonly, with headache; a minority of cases occur in middle-aged and elderly patients devoid of a prior seizure history (5, 6).Radiologically, PLNTY shows marked pleomorphism, presenting as well-circumscribed solid/cystic lesions or infiltrative masses. CT typically reveals nodular lesions with irregular gravel-like calcifications and variable margins (well-demarcated or ill-defined). On MRI, lesions are characteristically hypo- or isointense on T1WI, markedly hyperintense on T2WI/FLAIR, and consistently non-enhancing (1, 7). Key radiological hallmarks include ill-defined cortical masses with T2WI “pepper-and-salt sign” and CT gravel-like calcifications; extensive microcalcifications indicate a chronic progressive disease course. Notably, the transmantle sign—a classic marker of focal cortical dysplasia (FCD) reflecting cortical-white matter disorganization and drug-resistant epilepsy—has been reported in some PLNTY cases, all of which were pathologically confirmed to have concurrent FCD. This underscores the critical need to differentiate PLNTY from FCD in clinical diagnosis and management (4).

For a calcified, hypointense, non-enhancing temporal lobe lesion associated with long-standing drug-resistant epilepsy, the differential diagnosis includes cavernous malformation, DNET, ganglioglioma, oligodendroglioma, meningioangiomatosis, CAPNON, and, if extra-axial, calcified meningioma. FCD may be considered in epilepsy surgery, but calcification is atypical; typical MRI findings are cortical thickening, gray-white blurring, and/or a transmantle sign (8). Oligodendroglioma is molecularly defined by IDH mutation and 1p/19q codeletion; enhancement is variable, so absence of enhancement does not exclude it (9). DNETs are cortical, epilepsy-associated glioneuronal tumors, often cystic or multinodular rather than defined by a “triangular sign (10). Meningioangiomatosis has variable immunophenotype, so EMA/CD34 should not be overinterpreted (11). CAPNON is a rare but important calcified epilepsy-associated mimic (12).

Histopathologically, the most prominent hallmarks of PLNTY lesions are infiltrative growth, the presence of oligodendroglioma-like cellular components, extensive calcification, and diffuse immunoreactivity for CD34 (13). Activation of the mitogen-activated protein kinase (MAPK) pathway—particularly via the BRAF V600E mutation—or translocations involving fibroblast growth factor receptor 2 and 3 (FGFR2/FGFR3) constitutes another key characteristic molecular alteration (1, 9). Notably, PLNTY lesions rarely harbor isocitrate dehydrogenase (IDH) mutations or 1p/19q codeletion (14). Analyses have shown that BRAF V600E-positive PLNTY exhibits a more indolent clinical course, with clinical symptoms emerging only at an advanced stage and recurrence being uncommon (15, 16). Moreover, the expression of mutant BRAF proteins may disrupt neural networks, leading to abnormal neuronal discharges in different regions and thereby contributing to diverse seizure phenotypes. BRAF V600E is a constitutively active kinase in the MAPK pathway, while FGFR2/3 fusion proteins activate downstream pathways such as MAPK and PI3K-AKT through ligand-independent dimerization, rendering these molecules potential therapeutic targets (17),Importantly, in previously reported PLNTY cases, BRAF mutations and FGFR2/FGFR3 mutations exhibit genetic exclusivity—i.e., these two types of mutations do not coexist in the same case—suggesting that they may act as independent driver events in tumorigenesis (5, 16, 18–20). Notably, there was a case of a pediatric patient diagnosed with PLNTY harboring an FGFR3-TACC3 (Transforming Acidic Coiled-Coil Containing Protein 3) fusion who experienced recurrence 17 months postoperatively. MRI revealed a 4.1 cm partially solid and cystic lesion in the left temporal lobe accompanied by extensive edema, and postoperative pathology confirmed the recurrence as glioblastoma (21). Furthermore, the molecular subtyping characteristics of PLNTY are closely associated with its prognosis, conferring significant value for prognostic stratification.

This case aligns with the core clinical-pathological features of PLNTY: First, anatomically and clinically, the lesion arises in the left temporal pole (the most common site for PLNTY), and the patient presented with 6-year drug-resistant epilepsy progressing from focal facial twitching to mixed seizures, consistent with its definition as a long-term epilepsy-associated tumor. Second, pathologically and molecularly, the lesion showed thin-walled vascular clusters, hyaline degeneration, calcification and glial proliferation; immunohistochemistry revealed diffuse CD34 positivity, GFAP(+), Olig-2(+), IDH-1 R132H (–), and a low Ki-67 index of 1%. Molecular testing confirmed weak BRAF V600E positivity, consistent with the characteristic MAPK pathway activation in PLNTY. Third, therapeutically and prognostically, complete seizure control was achieved after lesionectomy, matching the indolent biological behavior and excellent prognosis of PLNTY following gross total resection.

Notably, this case presents atypical imaging features. While typical PLNTY shows T2WI/FLAIR hyperintensity (with occasional “pepper-and-salt sign”), our patient demonstrated T2WI/FLAIR hypointensity. Pathological correlation indicates this atypical signal arises from extensive intralesional calcification—calcified foci characteristically appear hypointense on T2WI, and the 6-year disease course provided sufficient time for this chronic calcification to develop. The initial misdiagnosis of cavernous hemangioma (which also shows T2WI hypointensity due to hemosiderin deposition) highlights the radiological non-specificity of PLNTY, emphasizing the need for vigilance in evaluating hypointense temporal lobe lesions with calcification.

DNA methylation profiling was not performed in this case, which represents a limitation of this report. Although the diagnosis was supported by integrated histopathological, immunohistochemical, and molecular findings, methylation-based classification has become increasingly important in CNS tumor diagnostics and may improve diagnostic precision, particularly for rare or histologically overlapping low-grade neuroepithelial tumors (22). In PLNTY, methylation profiling may help further distinguish this entity from other epilepsy-associated low-grade neuroepithelial tumors and refine integrated diagnosis Future cases may benefit from DNA methylation analysis when tissue and resources are available.

This case has two key clinical implications. First, it refines the diagnostic algorithm for atypical PLNTY: for hypointense temporal lobe lesions with calcification, no enhancement, and long-standing drug-resistant epilepsy, clinicians should maintain high suspicion for PLNTY even if cavernous hemangioma is initially suspected. Prompt pathological and molecular testing (focused on BRAF, FGFR2 and FGFR3) is critical to avoid diagnostic delays or overtreatment (asymptomatic cavernous hemangiomas may be managed conservatively, while PLNTY requires surgical resection). Second, it underscores the role of molecular subtyping in personalized follow-up: BRAF V600E-mutant PLNTY carries low recurrence risk and warrants routine surveillance, whereas FGFR2/FGFR3 fusion subtypes require prolonged, more frequent imaging to detect potential malignant progression.

## Conclusion

This case reports an adult with temporal pole PLNTY manifested as long-standing drug-resistant epilepsy. Atypical T2WI/FLAIR hypointensity caused by extensive calcification easily leads to misdiagnosis as cavernous malformation. Definite diagnosis was established by combined histopathology, immunohistochemistry and BRAF V600E detection. Gross total resection achieved complete seizure control and favorable prognosis. Clinicians should expand the differential diagnosis for calcified non-enhancing temporal lobe lesions associated with refractory epilepsy, and emphasize integrated pathological and molecular assessment. DNA methylation profiling is recommended for diagnostically challenging PLNTY cases in future clinical practice.

## Data Availability

The original contributions presented in the study are included in the article/supplementary material. Further inquiries can be directed to the corresponding authors.
